# *Dirofilaria* in Humans, Dogs, and Vectors in Austria (1978–2014)—From Imported Pathogens to the Endemicity of *Dirofilaria repens*

**DOI:** 10.1371/journal.pntd.0004547

**Published:** 2016-05-19

**Authors:** Hans-Peter Fuehrer, Herbert Auer, Michael Leschnik, Katja Silbermayr, Georg Duscher, Anja Joachim

**Affiliations:** 1 Institute of Parasitology, Department for Pathobiology, University of Veterinary Medicine Vienna, Austria; 2 Institute of Specific Prophylaxis and Tropical Medicine, Medical University Vienna, Austria; 3 Small Animal Clinic, Department for Companion Animals, University of Veterinary Medicine Vienna, Vienna, Austria; University of Würzburg, GERMANY

## Abstract

**Background:**

*Dirofilaria repens* and *D*. *immitis* are filarioid helminths with domestic and wild canids as main hosts and mosquitoes as vectors. Both species are known to cause zoonotic diseases, primarily pulmonary (*D*. *immitis*), ocular (*D*. *repens*), and subcutaneous (*D*. *repens*) dirofilariosis. Both *D*. *immitis* and *D*. *repens* are known as invasive species, and their distribution seems associated with climate change. Until very recently, both species were known to be nonendemic in Austria.

**Methodology and Principal Findings:**

Metadata on introduced and possibly autochthonous cases of infection with *Dirofilaria* sp. in dogs and humans in Austria are analysed, together with analyses of mosquito populations from Austria in ongoing studies.

In Austria, most cases of *Dirofilaria* sp. in humans (30 cases of *D*. *repens*—six ocular and 24 subcutaneous) and dogs (approximately 50 cases—both *D*. *immitis* and *D*. *repens*) were most likely imported. However, occasionally infections with *D*. *repens* were discussed to be autochthonous (one human case and seven in dogs). The introduction of *D*. *repens* to Austria was confirmed very recently, as the parasite was detected in Burgenland (eastern Austria) for the first time in mosquito vectors during a surveillance program. For *D*. *immitis*, this could not be confirmed yet, but data from Germany suggest that the successful establishment of this nematode species in Austria is a credible scenario for the near future.

**Conclusions:**

The first findings of *D*. *repens* in mosquito vectors indicate that *D*. *repens* presumably invaded in eastern Austria. Climate analyses from central Europe indicate that *D*. *immitis* also has the capacity to establish itself in the lowland regions of Austria, given that both canid and culicid hosts are present.

## Introduction

Various vector-borne helminths are prevalent in Europe, including those transmitted by mosquitoes, such as *Dirofilaria repens* and *D*. *immitis* (*Spirurida onchocercidae*) (Table 1) [1]. The most important definitive hosts of *D*. *repens* are dogs, but the parasite can also infect wild carnivores like red foxes and wolves as well as cats and humans [2]. It is the causative agent of subcutaneous and ocular dirofilariosis. The distribution of *D*. *repens* is limited to the Old World, with highly prevalent areas (prevalences in dogs of >10%) in southern and eastern Europe, Asia Minor, central Asia, and Sri Lanka [3]. More than 1,500 cases of human subcutaneous or ocular dirofilariosis caused by this pathogen have been documented worldwide [3–5]. However, the estimated number of unreported cases is probably much higher [6]. Compared to *D*. *immitis*, the infestation with *D*. *repens* is less severe, with subcutaneous nodules that can be excised surgically.

**Table 1:** Comparison of *Dirofilaria repens* and *D*. *immitis*.

*Dirofilaria repens*	*Dirofilaria immitis*—canine heartworm
• Canine and feline subcutaneous dirofilariosis	• Canine and feline cardiopulmonary dirofilariosis
• Zoonotic pathogen—human subcutaneous and ocular dirofilariosis	• Zoonotic pathogen—human pulmonary and ocular dirofilariosis
• Poor mammalian host specificity, with Canidae and Felidae as final hosts	• Poor mammalian host specificity, with Canidae and Felidae as final hosts
• Human—less suitable hosts	• Human—less suitable hosts
• Wild mammalian hosts: foxes	• Wild mammalian hosts: foxes, jackals, wolves, and pet ferrets
• Distribution: limited to Old World—southern and eastern Europe, Asia Minor, central Asia, and Sri Lanka	• Distribution: temperate, tropical, and subtropical areas of the world (Europe: main distribution in Mediterranean regions)

*D*. *immitis* is responsible for canine and feline cardiopulmonary dirofilariosis [7]. Canine cardiopulmonary dirofilariasis, or heartworm disease, is a potentially life-threatening disease caused by adult *D*. *immitis* filariae [7]. Besides dogs, cats, ferrets, and wild carnivores (e.g., foxes, jackals, and wolves) may also serve as definite hosts of *D*. *immitis* but are asymptomatic in most cases [8]. Cats are generally more resistant to adult *Dirofilaria*, showing no or only nonspecific clinical signs [3].

As a zoonotic agent, *D*. *immitis* is the causative of human pulmonary dirofilariasis, but this parasite was recently also associated with ocular dirofilariasis [3,6]. However, humans are less suitable hosts, and the parasite usually cannot complete its life cycle. It induces local inflammation and granuloma formation in its human host without reaching maturity. *D*. *immitis* is distributed in temperate, tropical, and subtropical areas of the world. In Europe, the main distribution is located in Mediterranean regions, where high prevalences in dogs are observed, and in several areas, both *D*. *repens* and *D*. *immitis* coexist (e.g., [4]). In untreated dogs, heartworm prevalence rates ranging from 50% to 80% were reported in the Po Valley area in Italy (reviewed in [7]). Thirty-three cases of human pulmonary dirofilariosis had been documented in Europe by 2012, but as with *D*. *repens*, the true number of cases is assumed to be considerably higher.

In central Europe (including Austria, the Czech Republic, Germany, Hungary, Liechtenstein, Poland, Slovakia, Slovenia, and Switzerland; definition of central Europe according to the World Fact Book: https://www.cia.gov/Library/publications/the-world-factbook/fields/2144.html), the first cases of *D*. *immitis* were described in four dogs in Switzerland in 1995 [9]. The first potential autochthonous findings of *D*. *repens* north of the Alpine Arc were documented in 11 clinically asymptomatic dogs from the south of Switzerland in 1998 [10]. *D*. *repens* and *D*. *immitis* have been documented more frequently in recent years, and autochthonous findings in dogs as well as mosquitoes are reported in new areas where both filarioid species were not known as endemic before [3,7,11]. By now (with the exception of Liechtenstein), both parasites have been reported in all countries neighboring Austria (Germany, the Czech Republic, Slovakia, Hungary, Slovenia, Italy, and Switzerland) [3,7,11–17], and it is obvious that *D*. *repens* was documented in most central European areas prior to *D*. *immitis*.

Within this article, the authors describe the findings of imported and (potentially) autochthonous findings of *D*. *repens* and *D*. *immitis* in dogs, humans, and mosquitoes in Austria.

## Methods

**Fig 1:**
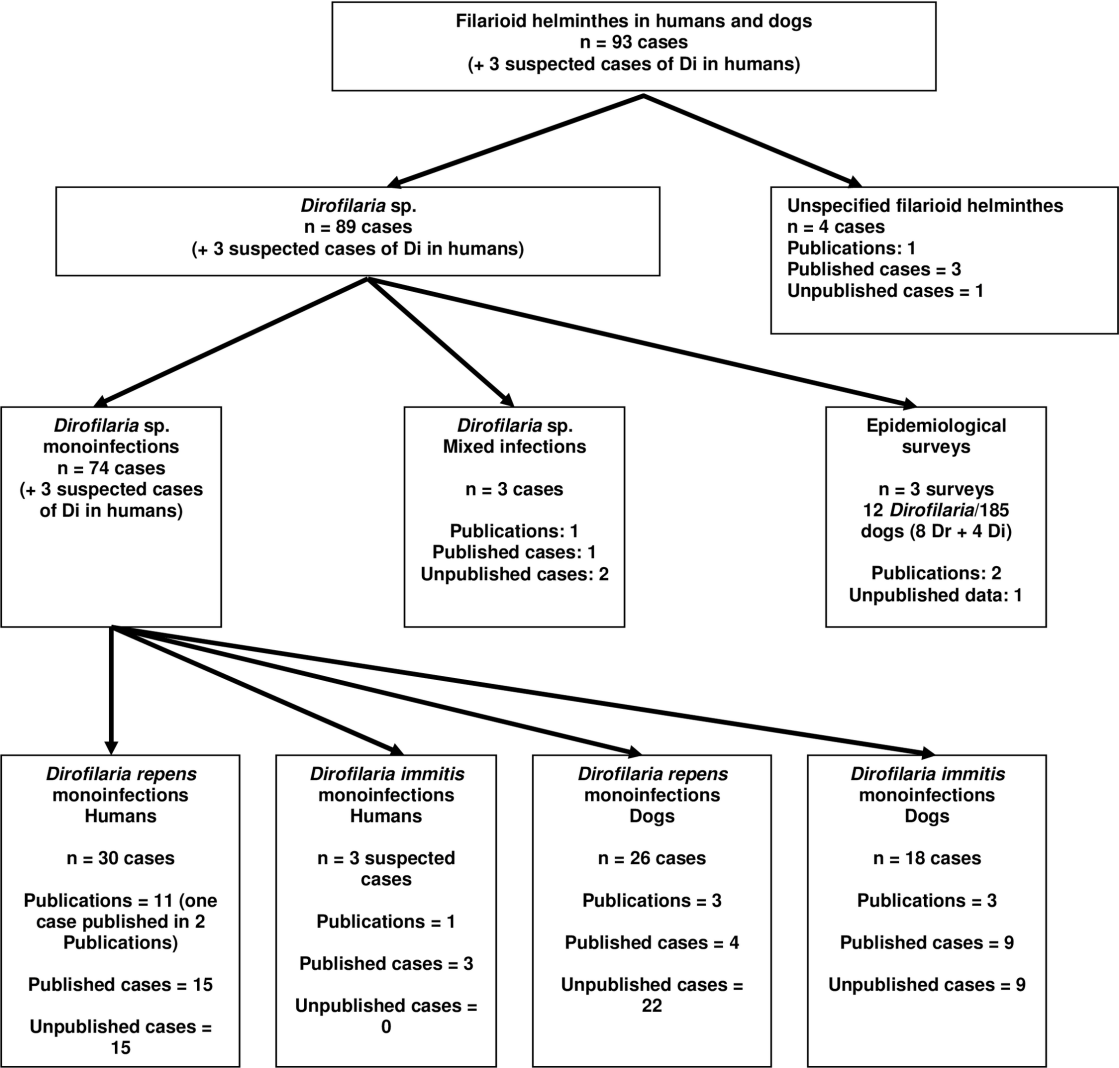
Flow chart (Di = *Dirofilaria immitis*).

Published as well as unpublished cases of imported and autochthonous documentations of *D*. *repens* and *D*. *immitis* in dogs (definite hosts), humans (accidental hosts), and mosquitoes (vectors) in Austria are summarized (Fig 1). Published cases were examined using electronically available databases (NCBI, Scopus, Google Scholar) with the keywords “Dirofilaria” and “Austria.” Literature published in German was examined in the same way. Furthermore, unpublished cases were examined in electronic patient databases for *Dirofilaria* spp. (dogs: University of Veterinary Medicine Vienna; humans: Medical University of Vienna).

## Epidemiology of *Dirofilaria* spp. in Austria

### Human Dirofilariosis in Austria

#### D. repens

Since 1978, 30 cases of human dirofilariosis caused by *D*. *repens* were reported in Austria, 16 (53%) in male patients and 14 (47%) in females (Table 2). Of these, 24 (80%) were subcutaneous infections and six (20%) ocular lesions. All patients presented clinical symptoms (appearance of skin nodes or wandering skin pain). Twenty-seven patients reported travel activity to at least one country known to be endemic for *D*. *repens* prior to infection. Fifty-three percent of all patients had been to the Mediterranean region, 13% to Hungary, and 27% overseas. About 30% of these infections were estimated to have been acquired in neighboring countries of Austria.

**Table 2:** Documented human *Dirofilaria* cases in Austria. Dr: *Dirofilaria repens*; Di: *Dirofilaria immitis*; f: female; m: male; +: positive;–: negative; nd: not determined; His: Histology; Ser: Serology; maf: microfilariae (adult stage); mif: microfilariae (first larval stage); Eos: eosinophils; IgE: immunoglobulin E; Dv: *Dipetalonema viteae* antigen.

Number	Year	Sex	Age	Pathology and/or Organ	Diagnosis	Geographical Anamnesis	Reference (if published)
1	1978	f	39	hip, upper leg, knee	His: Dr (maf); Ser: Di–; IgE: 100 IU	Greece	Bardach et al. 1981[25]
2	1989	f	27	upper eyelid	His: Dr (maf); Ser: Di –	East Asia	Lammerhuber et al. 1989 [26]
3	1992	m	36	head (occipital)	His: Dr (maf)	Hungary, Greece, Italy	Auer 2004 [19]
4	1995	m	44	linea axillaris anterior (right)	His: Dr (maf)	Bahrain, Greece	Schuller-Petrovic et al. 1996 [27]
5	1995	m	45	lower leg (left)	His: Dr (mif)	nd	Bischof et al. 2003[28]
6	1996	m	35	epididymis	His: Dr (maf); Eos: 10%; Ser: Di +, Dr: +	Italy, Portugal	Auer et al. 1997 [29]
7	1998	f	61	orbital cavity	His: Dr (maf); Ser: Di –	Italy, Greece	Braun et al. 1999 [30] Groell et al. 1999 [31]
8	1997	f	23	shoulder	His: Dr (maf); Ser: Dv –	Bosnia	Auer et al. 2004 [19]
9	1998	m	23	right inguinal lymph nodes	His: Dr (maf); Ser: Di +, Dr +; Eos: 8%	Slovenia, Spain, Albania	Auer et al. 2004 [19]
10	1998	f	48	left chest, left axillary region	His: Dr (maf); Ser: Di +	Spain, Greece (Korfu)	Auer et al. 2004 [19]
11	1998	m	43	sacral	His: Dr (maf); Ser: Di +	Malta, Portugal, Italy (Sardinia)	Auer et al. 2004 [19]
12	1999	m	4	neck, back	Ser: Dr +; IgE: >1,000 IU; Eos: 10%	Italy	^a^
13	2000	m	59	right cheek	Ser: Dr +; IgE: 1,000 IU	nd	^a^
14	2000	f	37	right chest	His: Dr (maf); Ser: Dr +	Turkey, Spain	Auer et al. 2004 [19]
15	2001	m	11	inguinal lymphoma	Ser: Dr +, Di +	Indonesia (Bali)	^a^
16	2001	f	60	eye	His: worm not specified; Ser: +	nd	^a^
17	2002	m	42	upper extremity	His: Dr (maf); Eos: 6%; Ser: Dr +, Di +	Ethiopia, Ghana	^a^
18	2003	m	61	skin (wandering knot)	Ser: Dr–, Di +	Peru	^a^
19	2003	m	54	inguinal hernia	Ser: Dr +, Di +	nd	^a^
20	2006	f	34	palm	His: Dr (maf); PCR Dr; Ser: Dr +, Di -	Austria (Nickelsdorf, Burgenland)	Auer and Susani (2008) [18]
21	2008	f	62	cheek, oral mucosa	His: Dr (maf); PCR Dr	Hungary	^a^
22	2009	m	61	epididymis	His: Dr (maf); PCR Dr; Eos: 9%	Namibia	^a^
23	2009	f	53	right chest	His: Dr (maf); PCR Dr	Italy	Böckle et al. 2010 [32]
24	2011	f	46	eye (subconjunctival)	His: Dr (maf); PCR Dr	Bosnia	Ritter et al. 2012 [33]
25	2011	f	65	lumbar region	His: Dr (maf); PCR Dr	East Asia, Sri Lanka, India	^a^
26	2011	f	49	head (temporal)	His: Dr (maf)	Croatia, Serbia	^a^
27	2012	m	53	orbital cavity	His: Dr (maf); PCR Dr	Italy, Hungary, Croatia	^a^
28	2012	f	39	eye, migrating worm	His: Dr (maf)	India	^a^
29	2014	m	75	umbilical hernia	His: Dr (maf); PCR Dr	Hungary	^a^
30	2014	m	50	inguinal hernia	His: Dr (maf); PCR Dr	Greece	^a^

^a^Unpublished patient data archived at the Medical University Vienna.

Only one human case of subcutaneous dirofilariosis was discussed as autochthonous acquired infection [18]. In September 2006, a 34-year-old border police officer from Nickelsdorf (Burgenland) presented a “tumor” 1 cm in diameter on the right palm of her hand after a mosquito bite. Histology and PCR were positive for *D*. *repens* and gave a negative result for *D*. *immitis*. Although the woman mentioned that she had never left Austria at geographical anamnesis, her occupation at the border to Hungary raised questions about the exact location where the infection was acquired.

#### D. immitis

Three suspected cases of human pulmonary dirofilariosis cases caused by *D*. *immitis* have been observed in Austria so far [19]. Patients presented pulmonary symptoms and were positive for *D*. *immitis* at serology. Because of the location in the lung, no invasive biopsies were conducted to confirm the presence of the parasite.

### *Dirofilaria* Infections in Dogs in Austria

#### D. repens

**Fig 2:**
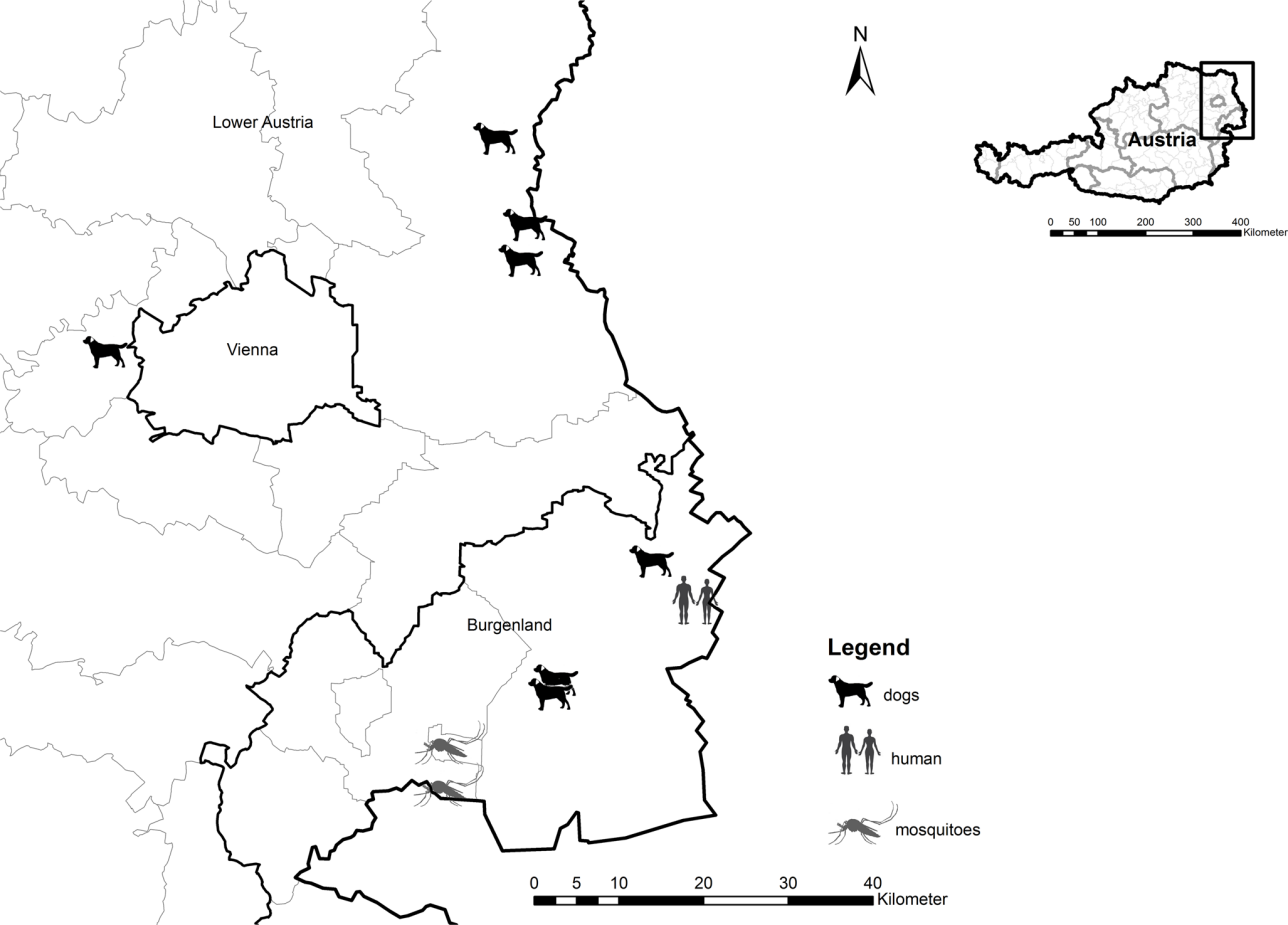
Potential autochthonous cases of *D*. *repens* in humans, dogs, and mosquitoes in eastern Austria.

Overall, *D*. *repens* was detected in 37 dogs in Austria (Table 3). Excluding epidemiological surveys and mixed infections with *D*. *immitis*, 26 dogs presented monoinfections. Ten (38%) dogs were female and 16 were male (62%). In only five cases (19%) of the monoinfections did symptoms like skin nodules lead to the diagnosis of dirofilariosis. In all other cases, the pathogens were found incidentally or during travel screening. Fifteen (58%) of the dogs infected with *D*. *repens* (including mixed infections with *D*. *immitis*) had previous travel activity to countries neighboring Austria known to be endemic for *D*. *repens* (Hungary, Slovakia, or Germany). Seven (27%) of the monoinfections were reported in Austrian dogs whose travel activity remained unclear. However, an epidemiological study conducted in eastern Austria documented the findings of microfilariae of *D*. *repens* in one of eight dogs in Gänserndorf (Lower Austria) and six of 90 dogs in Neusiedl (Burgenland) by PCR and Knott test [20]. All potentially autochthonous cases (with no reported time spent abroad) were reported from eastern Austria (Lower Austria and Burgenland) only (Fig 2). With the exception of one case in Gablitz (west of Vienna), all of those cases were documented at the border areas to Slovakia and Hungary.

**Table 3:** Documented cases of *Dirofilaria repens* and *Dirofilaria immitis* in dogs in Austria. +: positive;–: negative; nd: not determined; His: Histology; Ser: Serology; Maf: microfilariae (adult stage); mif: microfilariae (first larval stage) in blood, if not specified otherwise; Eos: eosinophils; IgE: immunoglobulin E; Ag-ELISA: SNAP Canine Heartworm Antigen Test Kit (IDEXX Laboratories).

Number	Year	Sex (*neutered*)	Age	Breed	Symptoms and/or Reason for examination	Diagnosis	Geographical Anamnesis	Therapy	Reference (if published)
***D*. *repens***									
1	before 2001	f *(n)*	4	mongrel	subcutaneous nodule	Maf +; mif +	Greece	–	Leschnik et al. (2008) [34]
2	before 2001	f *(n)*	1	Kuvasz crossbreed	hematuria,subcutaneous nodules (after primary diagnosis)	mif + in urine	Hungary	+	Kleiter et al. (2001) [35]
3	2002	f *(n)*	11.5	mongrel	incidental finding, tumor cytology	mif +	Hungary	–	^a^
4	2004	m	2	Dachshund	incidental finding, hematology	mif +; PCR +; Ag-ELISA –	Hungary	+	Leschnik et al. (2008) [34]
5	2007	f *(n)*	8	mongrel	conjunctivitis, swelling of the lower eye lid	Maf; mif +; Eos: 11%	Austria (Zurndorf, Burgenland)	+	^a^
6	2008	m	>3	mongrel	incidental finding	PCR +	Hungary	–	^a^
7	2008	m	>3	German Shorthaired Pointer	incidental finding	PCR +	Hungary, lives in district Neusiedl	–	^b^
8	2008	f	<3	German Wirehaired Pointer	incidental finding	PCR +	Slovakia, lives in district Neusiedl	–	^b^
9	2008	f	>3	Large Munsterlander	incidental finding	PCR +	Germany, lives in district Neusiedl	–	^b^
10	2008	f	>3	Golden Retriever	incidental finding	PCR +	Austria (Podersdorf; Burgenland)	–	^b^
11	2008	m	>3	Labrador Retriever	incidental finding	PCR +	Austria (Podersdorf; Burgenland), lives in district Neusiedl	–	^b^
12	2008	m	7	Wirehaired Dachshund	incidental finding	PCR +	Austria (Oberweiden; Gänserndorf), lives in district Gänserndorf	unclear	^b^
13	2008	m	10	German Wirehaired Pointer	incidental finding	PCR +	Austria (Zwerndorf; Gänserndorf), lives in district Gänserndorf	–	^b^
14	2008	m	8	Hanoverian Tracking Hound	incidental finding	PCR +	Hungary, lives in district Gänserndorf	–	^b^
15	2008	m	6	WireHaired Dachshund	incidental finding	PCR +	Slovakia, lives in district Gänserndorf	–	^b^
16	2008	f	4	Labrador Retriever	incidental finding	PCR +	Hungary, lives in district Gänserndorf	–	^b^
17	2008–2010	f	6	unknown	subcutaneous mandibular cyst	Maf; PCR +	Austria (Gablitz, Lower Austria)—exported to Germany	nd	Pantchev et al. (2011) [36]
18	2012	m *(n)*	3	mongrel	incidental finding, blood	mif +; PCR +; Ag-ELISA –	Slovakia	+	^a^
19	2013	m *(n)*	7	mongrel	incidental finding, tumor, cytology	mif +; PCR +	Slovakia	–	^a^
20	2013	m	3	mongrel	incidental finding, blood	mif +; PCR +; Ag-ELISA –	Romania	+	^a^
21	2013	m *(n)*	6	Golden Retriever	incidental finding, tumor, cytology	mif +; PCR +; Ag-ELISA –	Austria (Ebenthal/Lower Austria)	+	^a^
22	2013	f *(n)*	5	Newfoundland dog	incidental finding, tumor, cytology	mif +; PCR +	Hungary	+	^a^
23	2013	m *(n)*	2	mongrel	incidental finding, blood	mif +; PCR +; Ag-ELISA –	Croatia	+	^a^
24	2014	m *(n)*	5	Magyar Vizsla	incidental finding, tumor, cytology	mif +	Hungary	+	^a^
25	2014	m *(n)*	3	mongrel	incidental finding, subcutaneous nodule	mif +; PCR–; Ag-ELISA –	unclear	–	^a^
26	unclear	m *(n)*	5	mongrel	subcutaneous nodule	Maf; mif +	Hungary	+	^a^
***D*. *immitis***									
1	1985	m	4	Doberman	section	Two Maf in atrium cordis	former Yugoslavia, Italy, Greece, Turkey, France (Corsica)	–	Hinaidy et al. (1987) [37]
2	1987	nd	nd	Beagle	nd	nd	Japan, Saudi Arabia	nd	Löwenstein et al. (1988) [38]
3	1987	nd	nd	Rottweiler	nd	nd	Italy	nd	Löwenstein et al. (1988) [38]
4	1988	f	4	German Shepherd	necropsybloody expectoration, apathy, cough, dyspnea, inappetence, ascites	Approx. 40 Maf in right heart chamber and arteria pulmonalis	Italy	–	Löwenstein et al. (1988) [38]
5	before 2001	m *(n)*	5	Spaniel	travel screening	Ag-ELISA +; mif +	Spain	–	Leschnik et al. (2008) [34]
6	before 2001	f *(n)*	6	crossbreed	travel screening	Ag-ELISA +; mif +	Greece	–	Leschnik et al. (2008) [34]
7	before 2001	f *(n)*	3.5	Greyhound	travel screening	Ag-ELISA +; mif +	Spain	–	Leschnik et al. (2008) [34]
8	2001	f *(n)*	5	crossbreed	travel screening	Ag-ELISA +; mif	unclear	–	Leschnik et al. (2008) [34]
9	2002	m *(n)*	3	Boston Terrier	travel screening	Ag-ELISA +	United States of America (Florida)	–	Leschnik et al. (2008) [34]
10	2009	f *(n)*	6	Labrador Retriever	travel screening	Ag-ELISA +; mif	Greece	+	^a^
11	2009	m *(n)*	3.5	crossbreed	travel screening	Ag-ELISA +; mif +	Greece	+	^a^
12	2010	m	6	Rottweiler	incidental finding, hematology	Ag-ELISA +; mif + in blood and urine	Romania	–	^a^
13	2011	f *(n)*	5	Greyhound	travel screening	Ag-ELISA +; mif +	Spain	+	^a^
14	2011	m	5	Galgo Español	travel screening	Ag-ELISA +; mif +	Spain	+	^a^
15	2011	f *(n)*	5	Galgo Español	travel screening	Ag-ELISA +; mif +	Greece	+	^a^
16	2011	f *(n)*	1	Terrier	travel screening	Ag-ELISA +; mif	Serbia	+	^a^
17	2012	m	5	crossbreed	travel screening	Ag-ELISA +; PCR +; mif +	Croatia, southern France	+	^a^
18	2014	m *(n)*	4	crossbreed	apathy	Ag-ELISA +; PCR–; mif	Hungary	+	^a^
***Dirofilaria repens* and *D*. *immits***									
1	before 2001	f *(n)*	4	crossbreed	subcutaneous nodule	Ag-ELISA: +; Knott test: +; mif +; Maf *D*. *repens*	Greece	+	Kleiter et al. (2001) [35]
2	2014	m *(n)*	5	Magyar Vizsla	travel screening	Ag-ELISA +; PCR +; mif +	Hungary (East)	–	^a^
3	2014	m *(n)*	6	German Shepherd	travel screening	Ag-ELISA +; PCR +;mif +	Hungary (West)	+	^a^
**Epidemiological surveys**									
1	1999–2003	Five of 87 dogsFour cases *D*. *immitis*, one case *D*. *repens*			travel screening	Knott test, Ag-ELISA	Mediterranean		Prosl et al. (2003) [39]
2	2008	One of eight *D*. *repens*			epidemiological survey	Knott test, PCR	Austria (Lower Austria, Gänserndorf Distict)		Duscher et al. (2009) [20]
3	2008	Six of 90 *D*. *repens*			epidemiological survey	Knott test, PCR	Austria (Burgenland, Neusiedl District)		^a^
**Filaroid infection without species determination**									
1	before 2001	m	1	Kuvasz	travel screening	mif +	Hungary	–	Leschnik et al. (2008) [34]
2	2001	f *(n)*	8	crossbreed	travel screening	mif +	Greece	+	Leschnik et al. (2008) [34]
3	2001	m *(n)*	2	crossbreed	travel screening	mif +	Greece	+	Leschnik et al. (2008) [34]
4	2005	m	7	Pudelpointer	incidental finding, histology stomach	mif +	Slovenia	–	^a^

^a^Unpublished patient data archived at the University of Veterinary Medicine Vienna.

^b^Duscher and Feiler, unpublished data.

#### D. immitis

Filarioid helminths of the species *D*. *immitis* were documented in 25 dogs in Austria (Table 3). Of 16 dogs presenting monoinfections with *D*. *immitis* with known sex, eight were male and eight female. Clinical signs of imported dogs were in general occult or mild and resolved after adequate therapy. All cases of *D*. *immitis* in Austria were diagnosed during necropsy, travel screening, or routine veterinary examination with hematology, and 81% of the examined dogs had a history of travel activity to the Mediterranean region (e.g., Italy, Greece) prior to infection; no case was suspected to be an autochthonous infection from Austria.

#### Mixed infections and infections of unspecified filarioid helminths

Mixed *D*. *repens* and *D*. *immitis* infections were examined in three dogs, all of which had reported travel activity. Microfilariae were observed in the blood of four dogs, without further differentiation of the parasites to species level.

### Wildlife Hosts

Blood samples of foxes from eastern (District of Gänserndorf in Lower Austria; *n* = 36; unpublished data) and western Austria (Tyrol and Vorarlberg; *n* > 500; unpublished data) were screened for the presence of filarioid helminths. However, to date, neither *D*. *repens* nor *D*. *immitis* have been observed in Austrian foxes or other possible wild hosts.

### Mosquitoes

Various species of the genera *Aedes*, *Ochlerotatus*, *Culex*, *Culiseta*, *Coquillettidia*, and *Anopheles* are potential vectors of *Dirofilaria* spp. [3,7] in Europe. Several epidemiological studies have been conducted in Austria and neighboring countries; however, in most of these studies, DNA of the pathogens was examined in field-sampled mosquitoes that were classified to species level, and entire animals from the same sampling site and date were pooled. So, the vector competence of several mosquito species remains unclear because this cannot be confirmed if entire mosquitoes (including the abdomen) are used for molecular analysis.

Currently, 46 mosquito species are known to be present in Austria [17]. Of these, *Aedes vexans*, *Ae*. *albopictus* (no stable populations in Austria in 2014), *Culiseta annulata*, *Culex pipiens* complex, *Anopheles maculipennis* complex, *An*. *algeriensis*, and *Ochlerotatus caspius* are potential vectors for *D*. *repens* [3,12,17,21–23]. Potential vectors of *D*. *immitis* in Austria are *Coquillettidia richardii*, *Ae*. *albopictus*, *Oc*. *caspius*, *Ae*. *vexans*, *An*. *maculipennis* complex, *Cx*. *pipiens* complex, and *Cx*. *modestus* [3,7,14,17].

*D*. *repens* in Austria was detected in mosquitoes in 2012 for the first time in a nationwide mosquito surveillance and monitoring program [22]. DNA of *D*. *repens* was examined in pools of *An*. *algeriensis* in Rust and *An*. *maculipennis* complex in Mörbisch, both in the federal state of Burgenland, bordering Hungary (Fig 2). To date, these are the only findings of autochthonous *Dirofilaria* spp. in mosquitoes in Austria.

## Conclusions

According to Simon et al. (2012), the transmission of *D*. *repens* and *D*. *immitis* is limited to two main preconditions:

(i) the presence of one mosquito species capable of transmitting the parasites, and (ii) the presence of a minimum number of dogs infected with adult helminths that produce microfilariae.

In Austria, competent mosquito vectors for the transmission of both *D*. *repens* and *D*. *immitis* are part of the Austrian Culicidae species inventory. The two most common mosquito species in Austria, *Ae*. *vexans* and the *Cx*. *pipiens* complex, may readily act as potential vectors of these pathogens [21].

The number of infected dogs might be the limiting factor for the establishment of *Dirofilaria* in Austria. It is estimated that 581,000–600,000 dogs live in 511,000 households in Austria, with a human population of 8,579,747 (Statistik Austria: www.statistik.at, date: 01.01.2015). Most of these dogs are kept in the house; outdoor and/or kennel keeping is uncommon in this country. This circumstance might delay the introduction and establishment of the nematodes and might be the reason why they are to date not autochthonous in Austria, while this is the case for neighboring countries.

The above-mentioned preconditions are themselves influenced by several factors like human behavior with respect to pets (pet travel and health care) and wildlife (e.g., high fox populations after rabies eradication programs), globalization, and climatic factors [7]. Several models have shown that the expansion from southern to central and northern Europe (up to 50° N in the case of *D*. *immitis*) is most probable. Both the heartworm predictive model (based on growing degree days) and the *Dirofilaria* development units show parts of eastern Austria (the regions where most *D*. *repens* cases in dogs, the findings in mosquitoes, and the human case were documented) as suitable for the introduction and/or establishment of *D*. *repens* as well as *D*. *immitis* [24]. More detailed studies should reveal the exact area of predicted establishment, especially for the most westerly spread.

The (potential) autochthonous findings of *D*. *repens* in one human, seven dogs, and two mosquito species indicate that this parasite is becoming endemic and establishing itself in Austria. However, a stable establishment of *D*. *repens* is still to be seen, as all these cases were documented within a relatively short time span. *D*. *immitis* does not appear to be endemic in Austria, but with regard to observations in the neighboring countries (particularly Hungary and Slovakia) it will probably become established in the near future. Therefore, regular monitoring of the mosquito population as well as the wild carnivore population is of urgent need.

The first findings of *D*. *repens* in mosquito vectors indicate that *D*. *repens* presumably invaded into eastern Austria in recent times. Veterinarians and medical physicians should be aware of possible autochthonous cases with these neglected pathogens in Austria. However, further monitoring of mosquitoes is necessary to observe the expansion of the distribution of *D*. *repens* and the possible invasion by *D*. *immitis* in Austria.

**Key Learning Points**

- Autochthonous findings of *D*. *repens* in mosquitoes, as well as potential autochthonous cases in dogs and humans, suggest that this parasite is establishing itself in Austria.
- Until now, *D*. *immitis* is only associated with travel activity, and the parasite is not (yet) endemic in Austria.
- The increase of cases with both *D*. *repens* and *D*. *immitis* in dogs in recent years makes it clear that veterinarians should also consider these parasites in the diagnosis of Austrian dogs without prior travel activity.
- Mosquito surveillance and observation of canid wildlife hosts for the presence of *D*. *repens* and *D*. *immitis* is necessary to evaluate the possible establishment of both parasites in the future.

**Top Five Papers**

1. Genchi C, Kramer LH, Rivasi F (2011) Dirofilarial infections in Europe. Vector Borne Zoonotic Dis 11:1307–17. doi: 10.1089/vbz.2010.0247.
2. Morchón R, Carretón E, González-Miguel J, Mellado-Hernández I (2012) Heartworm Disease (Dirofilaria immitis) and Their Vectors in Europe—New Distribution Trends. Front Physiol 3:196. doi: 10.3389/fphys.2012.00196.
3. Otranto D, Dantas-Torres F, Brianti E, Traversa D, Petrić D, et al. (2013) Vector-borne helminths of dogs and humans in Europe. Parasit Vectors 6:16. doi: 10.1186/1756-3305-6-16.
4. Silbermayr K, Eigner B, Joachim A, Duscher GG, Seidel B, et al. (2014) Autochthonous Dirofilaria repens in Austria. Parasit Vectors 7:226. doi: 10.1186/1756-3305-7-226.
5. Simón F, Siles-Lucas M, Morchón R, González-Miguel J, Mellado I, et al. (2012) Human and animal dirofilariasis: the emergence of a zoonotic mosaic. Clin Microbiol Rev 25:507–44. doi: 10.1128/CMR.00012-12.
